# Concurrent Quantification of Deoxynivalenol, Its Derivatives, and Nivalenol in Pet Food Using QuEChERS Combined with LC-MS/MS

**DOI:** 10.3390/toxins17120590

**Published:** 2025-12-10

**Authors:** Chae-Eun Yeo, Subin Gwon, Eun Hee Chang, Hyo Young Kim, Sung-Youn Kim, Kangmin Seo, Ji Hye Lee, Hyunjeong Cho

**Affiliations:** Experiment Research Institute, National Agricultural Products Quality Management Service, Gimcheon-si 39660, Republic of Korea

**Keywords:** deoxynivalenol, nivalenol, pet food, LC-MS/MS, QuEChERS, deoxynivalenol-3-glucoside

## Abstract

In the current research, we optimized a simultaneous method for quantifying deoxynivalenol (DON) and its derivative forms, deoxynivalenol-3-glucoside (D3G), 3-acetyl-deoxynivalenol (3-AcDON), 15-acetyl-deoxynivalenol (15-AcDON), and nivalenol (NIV), in pet food using QuEChERS combined with liquid chromatography quadrupole mass spectrometry. The developed method’s linearity, sensitivity, selectivity, accuracy, and precision were also validated. The limits of detection and quantification for this analysis method were 6.7–9.4 ng g^−1^ and 20.1–28.1 ng g^−1^, respectively. The average recovery (60.1–107.2%, RSD ≤ 9.3%) met the recovery (60–110%) and precision (RSDr ≤ 20%) criteria for DON specified in Commission Regulation (EC) No. 401/2006. A total of 246 pet food samples (68 cat and 178 dog food samples) collected in South Korea were analyzed. DON was detected in 11.8% of cat food and 8.4% of dog food samples, with concentrations ranging from 122.9 to 799.4 ng g^−1^ and 79.7 to 698.0 ng g^−1^, respectively. The co-occurrence rate of DON and its metabolites was 7.3% in dog food and 10.3% in cat food. NIV was not detected in cat food samples but was detected in two (1.1%) dog food samples at 23.4 and 32.0 ng g^−1^ contamination levels. All detected levels were below the regulatory guidance limit. Investigations of the effect of DON contamination levels according to the grain content of pet food revealed that the DON detection rate tended to increase with grain content. This study can be effectively utilized in quality control laboratories for high-throughput routine analysis of mycotoxins.

## 1. Introduction

Mycotoxins are secondary substances produced by fungi that inadvertently contaminate food and feed. These toxins can harm human and animal health and can be found in crops or stored foods. Over 400 mycotoxins have been identified, with aflatoxin, trichothecenes, fumonisin, ochratoxin A, and zearalenone being the most significant [1]. Type B trichothecenes, such as deoxynivalenol (DON), 3-acetyl-deoxynivalenol (3-AcDON), 15-acetyl-deoxynivalenol (15-AcDON), and nivalenol (NIV), are mainly generated by strains of *Fusarium culmorum* and *Fusarium graminearum* [2]. Among type B trichothecenes, DON is one of the most frequently encountered mycotoxins worldwide [3]. Christiane et al. [4] reported that over 10 years, DON emerged as the most prevalent mycotoxin in East Asia, including Korea, when dividing the occurrence of mycotoxins worldwide by region.

The toxic effects of DON in animals include vomiting, anorexia, growth retardation, immunotoxicity, reproductive and developmental disorders, altered neuroendocrine signaling, induction of proinflammatory genes, and altered intestinal integrity [5].

DON commonly appears in grains, which are used as feed ingredients in its conjugated (deoxynivalenol-3-glucoside [D3G]) and acetylated forms (3-AcDON and 15-AcDON) bound to sugars as a result of natural detoxification. 3- and 15-Ac-DON are primarily converted into DON through deacetylation in the body. D3G, which is commonly found in grains, is broken down into DON during the digestive process in the intestines of both animals and humans. As a result, these substances can significantly contribute to overall exposure to DON [6,7]. Due to these characteristics, assessing only the concentration of DON may underestimate the actual exposure risk. The European Food Safety Authority (EFSA) proposed a daily allowable intake of 1 ng/g body weight for the total amount of DON, 3-AcDON, 15-AcDON, and D3G in food [8]. The U.S. Food and Drug Administration (FDA) stipulates that animal feed should be produced with safety standards comparable to those for human food, ensuring it is free from harmful substances and accurately labeled [9]. In line with this global trend in food safety management, it is important to monitor not only DON but also its metabolites in pet food.

Commercial dog and cat foods are typically formulated with 30–50% grains [10], which include soybean, rice, corn, wheat, barley, and their by-products, providing inexpensive sources of energy and nutrients [11,12]. Cereal processing can concentrate mycotoxins, increasing the risk of contamination in cereal by-products used as feed ingredients [13,14]. However, very few studies have assessed the contamination levels of DON and its metabolites in commercial dog and cat foods. In 2017, EFSA [8] conducted a risk assessment on dog and cat food, including not only DON but also its metabolites, and reported that high concentrations of DON pose a potential risk of acute adverse effects in cats. Nevertheless, data on DON, 3-Ac-DON, 15-Ac-DON, and DON-3-glucoside in commercial pet foods were not measured directly but estimated from pet food formulations and the total concentrations of DON, 3-Ac-DON, 15-Ac-DON, and DON-3-glucoside in individual ingredients, which represents a limitation of the study [8].

The European Commission (EC) and the Ministry of Agriculture, Food and Rural Affairs (MAFRA) in South Korea set a recommended limit of 5000 ng g^−1^ for deoxynivalenol (DON), among type B trichothecenes, in feed for companion animals [15,16]. There are no regulatory limits for 3-AcDON, 15-AcDON, D3G, and NIV. Therefore, to ensure pet food safety and protect animal health, it is necessary to monitor not only DON but also D3G and other major type B trichothecenes in pet food, and efficient analytical methods are essential for such monitoring.

Recently, the QuEChERS extraction method combined with liquid chromatography quadrupole mass spectrometry (LC-MS/MS) has gained widespread use for extracting mycotoxin residues from animal feed, offering a highly efficient and effective approach for detecting these contaminants in various feed samples [17,18,19]. In 2021, the MAFRA of the Republic of Korea developed a QuEChERS-based LC–MS/MS method for the simultaneous quantification of seven major mycotoxins in animal feeds [20]. The sample preparation procedure was based on a QuEChERS approach. Samples were first extracted with 10% formic acid in water, followed by liquid–solid partitioning using anhydrous magnesium sulfate (MgSO_4_) and sodium chloride (NaCl). Subsequently, a dispersive solid-phase extraction (dSPE) step employing primary–secondary amine (PSA) and C18 sorbents was applied. The extract was then subjected to cold-induced precipitation at 4 °C for 30 min to remove residual matrix interferences [20]. Compared with conventional procedures previously used for feedstuffs, such as purification with SPE cartridges [21,22] or immunoaffinity columns (IACs) [23], followed by QuEChERS pretreatment and solvent evaporation/conversion steps [24], the present method is markedly simpler. It requires less preparation time and solvent while maintaining comparable recovery rates. In addition to, to minimize matrix effects in LC–MS/MS analysis and reduce the consumption of costly isotopically labeled internal standards, a post-ISTD injection strategy was implemented using an automated autosampler. This approach improved both analytical efficiency and reproducibility.

As a result, the analytical workflow was streamlined and rendered more cost-effective. The MAFRA method achieved a limit of quantification (LOQ) of 50 ng g^−1^ for deoxynivalenol, demonstrating sensitivity comparable to that of the U.S. FDA official method (C-003.03), which applies SIDA coupled with LC–MS/MS for multi-mycotoxin determination [25].

However, this method has not been applied to the determination of deoxynivalenol metabolites and nivalenol. To the best of our knowledge, studies on deoxynivalenol metabolites have primarily focused on grains [18,22,26] and livestock feed [21], and their application to pet food has not yet been investigated.

Commercial dog and cat foods contain diverse coexisting components including high-protein and high-fat ingredients, Maillard reaction products, lipid oxidation products, peptides, flavor additives, and coating fats [27], resulting in substantially greater matrix interferences and structural complexity compared with cereal or protein-based livestock feed or simple cereal matrices. These characteristics can cause severe ion suppression or enhancement during the analysis of highly polar mycotoxins, thereby compromising quantitative accuracy.

In addition, 3-acetyl-DON and 15-acetyl-DON are positional isomers with identical molecular weights and polarity under conventional LC conditions, they frequently co-elute, presenting a significant analytical challenge to achieving adequate chromatographic separation [2,28].

This study aimed to optimize and verify a technique for simultaneously analyzing primary forms of type B trichothecenes (3-AcDON, 15-AcDON, DON, NIV, and D3G) in dry pet food using LC-ESI-MS/MS, applying the MAFRA pretreatment protocol for sample preparation. The established method was applied to 246 dry pet food samples (68 cat and 178 dog food samples) collected in Korea in 2024 to determine DON, 3-AcDON, 15-AcDON, D3G, and NIV concentrations. This work represents the first validated approach for type B trichothecene analysis in dry pet-food matrices and provides the first monitoring data for 3-AcDON, 15-AcDON, D3G, and NIV in commercial pet foods.

The monitoring data on DON and its metabolites and NIV obtained in this study can provide a scientific basis for establishing regulatory limits and quality control standards for mycotoxins in pet food. They may contribute to future policy development and risk management strategies.

## 2. Results and Discussion

In this study, the MAFRA QuEChERS procedure (Section 4.3) was applied, while the LC–MS conditions were optimized. The mobile phase was modified from the original ammonium formate–formic acid system to ammonium acetate–acetic acid to improve ionization efficiency, and the column was changed from C18 to C18-PFP to enhance the separation of polar analytes. DON was analyzed in ESI(–) mode due to improved sensitivity and signal stability, and new MRM transitions were established for 3-AcDON, 15-AcDON, D3G, and NIV.

### 2.1. LC-MS/MS Optimization

The mycotoxin analysis method was optimized as follows: 1 µg/mL standard solution was injected at a flow rate of 10 µL min^−1^ using water containing 5 mM ammonium acetate and 0.1% acetic acid (A) and methanol/water (95/5:*v*/*v*) with 5 mM ammonium acetate and 0.1% acetic acid (B) as the mobile phase. The optimal precursor ion was selected based on the results of the full-scan mass spectra obtained in both the positive and negative ionization modes. The selected precursor ions were optimized by adjusting the collision energy (CE) values to generate stable and sensitive product ions, from which the characteristic ions were selected for analysis using the Multiple Reaction Monitoring (MRM) method. The quantification ion with the greatest sensitivity among the product ions was selected, while the ions with the next highest sensitivity were used as confirmation ions for qualitative analysis (Table 1). While developing the detection method, the MS/MS conditions were optimized for each analyte, focusing on the fragmentation reactions and ionization modes (Table 1). Most analytes predominantly formed major ions in the form of [M + CH_3_COO]^−^. However, 15-AcDON produced an [M + NH_4_]^+^ adduct ion in ESI^+^ mode, which exhibited higher intensity than the [M + CH_3_COO]^−^ ion generated in ESI^−^ mode. Therefore, the [M + NH_4_]^+^ ion was selected for quantification and identification. Negative ionization was applied for most mycotoxins except 15-AcDON, and ^13^C_17_-15-AcDON [29].

**Figure 1:**
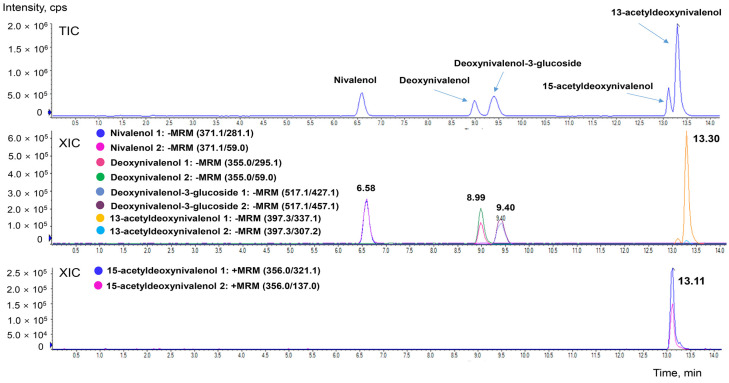
Total ion chromatograms (TIC) and extracted ion chromatograms (XIC) of deoxynivalenol, its derivatives, and nivalenol.

The chromatographic conditions enabled sufficient separation of the target mycotoxins within 15 min (Figure 1).

The analytes are highly polar compounds and include deoxynivalenol (DON, Log *p* = −0.72), 3-acetyl-deoxynivalenol (3-AcDON, −0.14), 15-acetyl-deoxynivalenol (15-AcDON, −0.69), deoxynivalenol-3-glucoside (D3G, −2.31), and nivalenol (NIV, −1.69). Based on the XLOGP3 model, their predicted Log *p* values range from −0.168 to −2.967, as obtained from SwissADME via MycoCentral [30]. The peak detection order in the LC analysis was NIV, DON, D3G, 15-AcDON, and 3-AcDON, showing a consistent correlation with the analytes’ polarity. Although D3G has the lowest Log *p* value, it was detected later than DON, which may be attributed to altered interactions within the column due to its glycosylated structure.

The chromatographic separation of the isomers 3-AcDON and 15-AcDON, which differ only in the position of the acetyl group, is a key challenge in this method. Since DON, 3-AcDON, and 15-AcDON produce identical product ions in MS/MS analysis, complete chromatographic resolution is essential for accurate quantification. Fiby et al. [31] evaluated 13 columns with varying physicochemical properties and manufacturers using water and acetonitrile (both containing 2 mM ammonium acetate) as mobile phases. The Acquity HSS T3 C18 column (2.1 mm × 100 mm, 1.8 μm; Waters) was deemed most suitable. However, D3G, DON, 15-AcDON, and 3-AcDON eluted rapidly at 1.70, 1.95, 3.23, and 3.31 min, respectively, resulting in inadequate retention. This rapid elution limits its applicability for complex feed matrices due to potential interferences. Panasiuk et al. [19] addressed this issue by employing a Luna Omega Polar C18 column (100 × 2.1 mm, 1.6 µm; Phenomenex, Torrance, CA, USA) with a mobile phase consisting of 0.2% acetic acid and acetonitrile. They completely separated 3-AcDON and 15-AcDON using a gradient from 15% to 18% ACN over 2–6 min. In this study, considering the high polarity of the analytes, a methanol–water mobile phase was used, supplemented with 5 mM ammonium acetate and 0.1% acetic acid to improve aqueous solubility and control retention. Previous studies, including those by Andrade et al. [32] and the EURL [29], successfully achieved the separation of 15-AcDON and 3-AcDON using C18 columns and a mobile phase consisting of water and MeOH containing 5 mM ammonium acetate. However, attempts to replicate these results under identical conditions in the present study were unsuccessful.

Consequently, a reversed-phase column modified with a pentafluorophenyl (PFP) functional group, which is known to provide enhanced separation of isomeric compounds, was employed to establish optimized analytical conditions. In addition, the two compounds exhibited distinct fragmentation patterns, allowing them to be distinguished by the mass spectrometer. In ESI^+^ mode, the precursor ion of 15-AcDON ([M + NH_4_]^+^, *m*/*z* 356.0) produced two characteristic product ions. The ion at m/z 321.00 was formed by the loss of the acetyl group at C-15 and the ammonium adduct, while the ion at *m*/*z* 137.0 originated from the fragmentation of the ring structure. These ions served as specific markers for the identification and quantification of 15-AcDON.

### 2.2. Performance of the Analytical Method

#### 2.2.1. Linearity

The calibration curve was prepared in a blank matrix with a series of concentrations: 10, 25, 50, 100, 250, 500 ng g^−1^. As shown in Table 2, the linearity of all five mycotoxins was excellent, with correlation coefficients (*R*^2^) in cat and dog samples being above 0.99.

#### 2.2.2. Analytical Method Limits of Detection and Quantification

The results showed that the LODs ranged from 6.7 to 9.4 ng g^−1^, and the LOQs ranged from 20.1 to 28.0 ng g^−1^ (Table 2). The proposed method achieved a lower LOQ for DON than the current Korea MAFRA method (50 ng g^−1^), U.S. FDA official method (C-003.03) demonstrating improved analytical sensitivity.

Zhao et al. [18] validated an LC–MS/MS method for seven mycotoxins in animal feed, reporting LODs of 5.0–5.9 ng g^−1^ for DON, 8.4 ng g^−1^ for NIV, 5.5–7.4 ng g^−1^ for D3G, 6.4–8.8 ng g^−1^ for 3-AcDON, and 5.3–10.0 ng g^−1^ for 15-AcDON. Sun et al. [33] reported LODs of 5.0 ng g^−1^ for DON, 2.0 ng g^−1^ for 15-AcDON, and 2.0 ng g^−1^ for 3-AcDON, with corresponding LOQs of 15, 5, and 5 ng g^−1^, respectively, in a QuEChERS-based cereal analysis. Zhao et al. [18] also presented low LODs in animal feed, reporting 5.0–5.9 ng g^−1^ for DON, 8.4 ng g^−1^ for NIV, 5.5–7.4 ng g^−1^ for D3G, 6.4–8.8 ng g^−1^ for 3-AcDON, and 5.3–10.0 ng g^−1^ for 15-AcDON. The LODs and LOQs obtained in the present study were higher than those in these previous reports [18,33], likely because both studies determined detection and quantification limits using S/N ratios of 3 and 10 directly measured from chromatograms.

This study evaluated quantification capability under actual analytical conditions based on the method detection limit, which reflects repeatability and recovery, rather than relying on the signal-to-noise (S/N) ratio method. This approach calculates LOD and LOQ using the slope of the calibration curve and the standard deviation of repeated measurements, thereby accounting for both instrument response and experimental variability throughout the analytical process.

#### 2.2.3. Intra and Interday Precision

The intra- and interday precisions (% relative standard deviation, [RSD]) were evaluated by spiking standards at 20 ng g^−1^ to assess the method’s precision. As presented in Table 2, the RSDr values for all samples were below 15%. The overall RSDr values were greater than the RSDr values, but remained at or approximately 20%; this suggests that the established method satisfies the requirements for determining multiple mycotoxins in pet food.

#### 2.2.4. Method Accuracy

The accuracy and precision of the method established in the current study were assessed based on the mean recovery and RSD of the spiking recovery test results, in which the matrix-matched external standard calibration method or isotopically labeled internal standard (ISTD) was applied for quantification.

**Figure 2:**
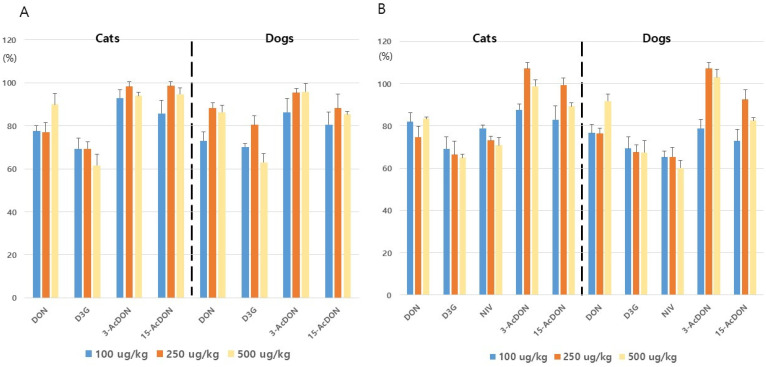
Mycotoxin recoveries obtained using the internal standard (**A**) and matrix-matched quantification method in dog and cat food sample (**B**). DON, deoxynivalenol; D3G, deoxynivalenol-3-glucoside; ActDON, acylated deoxynivalenol; NIV, nivalenol.

Three levels of mycotoxins were spiked into the blank matrix at concentrations of 100, 250, and 500 ng g^−1^, with three replicates for each level. Because the test method involved a 5-fold dilution, the concentrations observed in the instrument analysis were 20, 50, and 100 ng g^−1^. Figure 2 shows that recovery values ranged from 60.1 to 107.2%. All RSDs were within 9.3%, and the analysis was executed using the ISTD and matrix-matched calibration methods. In the analysis using ISTD, the recovery rates for dog and cat food samples, as shown in Figure 2A, were as follows: DON 73.1–89.9%, D3G 63.0–80.4%, 3-AcDON 86.3–98.3%, and 15-AcDON 80.6–98.6%, with an RSD of 1.7–6.6%. In the matrix-matched method, the recovery rates for dog and cat food samples were as follows: DON 74.7–91.8%, D3G 65.0–69.3%, 3-AcDON 78.8–107.2%, 15-AcDON 72.9–99.4%, and NIV 60.1–78.8%, with RSDs ranging from 0.7% to 9.3% (Figure 2B). This method satisfies the recovery and precision requirements specified in Commission Regulation (EC) No. 401/2006 [34] for DON at concentrations >100 ng g^−1^ and ≤500 ng g^−1^ (Recovery: 60–110%; RSDr ≤ 20%; RSDR ≤ 40%). In addition, the method meets the validation criteria outlined in SANTE/11813/2017 [35] including linearity, accuracy, precision, LOD, and LOQ and complies with the MAFRA [36] guidelines for feed analysis, including pet food.

The recoveries of DON, 15-AcDON, and 3-AcDON obtained in this study were comparable to those reported in previous studies on animal feed. Zhao et al. [18] extracted swine and poultry feed samples using an acetonitrile–water mixture followed by purification with MgSO_4_ and n-hexane, and reported recoveries ranging from 88.4% to 106.3%, which were similar to the results of the present study. Likewise, Panasiuk et al. [19] employed a Mycosep 225 clean-up column for swine and poultry feed and observed recoveries of 90–105%, showing a similar trend to our findings. In contrast, the recoveries of D3G in cat and dog feed, as well as those of NIV in dog feed, were somewhat lower than the values reported by Zhao et al. [18] (D3G: 79.0–107.1%; NIV: 79.6–93.3%) and Panasiuk et al. [19] (D3G: 92–96%; NIV: 94–106%).

In this study, the recoveries of D3G and nivalenol were approximately 60–70%. Although no official recovery criteria have been established for these analytes, quantitative analytical methods generally recommend recovery values above 70%. Therefore, further optimization is required to improve the recoveries of D3G and nivalenol. One possible approach to enhance recovery is the application of a standard addition calibration, in which standards are added during the sample preparation step. Moreover, although isotope-labeled internal standards were used in a post-ISTD manner in this study, spiking the internal standard (ISTD) prior to extraction could more effectively compensate for matrix-related losses and thereby improve analytical accuracy.

In addition, the accuracy of the proposed analytical method was evaluated through participation in the AAFCO International Proficiency Testing Program. As shown in Table 3, the ISTD experiment results showed an average value of 2033.1 ng g^−1^, with a *Z*-score of 0.63–0.78, and the matrix-matched experiment results exhibited an average value of 3163.8 ng g^−1^, with a *Z*-score of 0.65–0.77. In all cases, the *Z*-scores were considered satisfactory for both experiments. (|*Z*|: ≤ 2). The two methods did not significantly differ (*p* > 0.05). Therefore, both methods were demonstrated to be satisfactory.

#### 2.2.5. Matrix Effect

MEs frequently occur in mass spectrometry techniques such as LC-MS/MS, where the matrix of the sample co-elutes and affects instrument sensitivity owing to surface tension and viscosity at the ESI interface, leading to ion suppression or enhancement [37]. As shown in Figures S1–S4, both the matrix-matched calibration curves and the ISTD-corrected curves exhibited higher slopes than the solvent-based external calibration, indicating signal enhancement for all analytes. As shown in Table S1, in the ESI(−) mode, the matrix effect observed with the matrix-matched calibration was most pronounced for D3G in dog food (−134.9%), followed by DON (−94.2%), NIV (−93.4%), and 3-AcDON (−79.9%). In cat food, the matrix effect appeared in the order DON (−95.0%) > D3G (−94.3%) > NIV (−88.2%) > 3-AcDON (−82.1%).

In the ESI(-) mode, the matrix effect calculated using the ISTD-based approach was most pronounced for D3G in dog food (−130.7%), followed by 3-AcDON (−105.5%) and DON (−88.9%). In cat food, the largest signal enhancement was observed for DON (−128.4%), followed by 3-AcDON (−105.5%) and D3G (−92.3%). For NIV, no comparison could be made because a labeled internal standard was not available. In the ESI(+) mode, the matrix effect for 15-AcDON was −96.2% (dog) and −108.8% (cat) with the matrix-matched calibration, and −108.3% (dog) and −129.1% (cat) with the ISTD method. In dog food, the strongest signal enhancement was observed for the highly polar D3G, whereas in cat food, the least polar compound, 15-AcDON, showed the greatest enhancement.

The matrix effect showed similar results with both the matrix-matched and ISTD calibration methods. In general, signal enhancement arises when specific matrix components function as dopants, thereby improving the ionization efficiency of analytes with higher ionization energy [38]. In this study, the blank dog sample consisted primarily of protein- and carbohydrate-based ingredients, comprising chicken, defatted soybean meal, and rice flour, together with propylene glycol and glycerin. In contrast, the blank cat sample contained 75% fish ingredients, comprising herring, mackerel, cod, and merluza, and 25% fruits and vegetables, resulting in a matrix rich in fatty acids and phospholipids. These compositional differences between the blank matrices are presumed to influence ionization efficiency and consequently contribute to the observed differences in signal enhancement.

Without ISTD or matrix-matched calibration, analyte concentrations were overestimated by 1.8- to 2.4-fold relative to the true concentrations (Table S2). This highlights the necessity of applying appropriate matrix compensation, either matrix-matched or ISTD-based calibration, to achieve accurate quantification in complex pet food matrices.

### 2.3. Prevalence of the Deoxynivalenol, Its Derivatives, and Nivalenol in Dog and Cat Food

The QuEChERS-based extraction method established in this study, combined with isotope-labeled LC-MS/MS analysis, offers clear advantages regarding time and cost efficiency, making it particularly suitable for high-throughput laboratories dealing with complex pet food matrices. Accordingly, this validated analytical method was applied to monitor mycotoxin contamination in animal feed products distributed in the Korean domestic market.

In total, 246 pet food samples (68 cat and 178 dog food samples) were collected in 2024. Of these, 62 samples (25.2%) contained at least one mycotoxin concentration exceeding the LOQ. 

As illustrated in Table 4, DON was detected in 8 (11.8%) of the 68 cat food samples, with concentrations ranging from 122.9 to 799.4 ng g^−1^ and a median level of 268.9 ng g^−1^. In dog food, DON was detected in 15 (8.4%) of the 178 samples, with concentrations ranging from 79.7 to 698.0 ng g^−1^ and a median level of 317.5 ng g^−1^. The detection rate and concentration of DON were lower, to a small extent, in dog food than in cat food. 15-AcDON was detected in 5.6% of 178 dog food samples (10 samples) at concentrations varying from 22.5 to 207.9 ng g^−1^. Six out of sixty-eight contaminated (8.8%) cat samples contained 15-AcDON, with levels ranging from 33.8 to 101.6 ng g^−1^ and a median value of 58.2 ng g^−1^. NIV was not detected in cat food samples but was detected in two (1.1%) dog food samples at 23.4 and 32.0 ng g^−1^ contamination levels.

When compared with international regulatory and guidance values (Table S3), the DON concentrations observed in this study (79.7–799.4 ng g^−1^) were several to several tens of times lower than the established limits (1000–5000 ng g^−1^).

The EU and the MAFRA have set guideline levels of 5000 ng g^−1^ for dog and cat feed, whereas Japan enforces legislated compositional standards of 2000 ng g^−1^for dogs and 1000 ng g^−1^ for cats [39]. In the United States, the Food and Drug Administration (FDA) recommends that grains and grain by-products used in animal feed contain no more than 5000 ng g^−1^ of DON, with the additional recommendation that such ingredients not exceed 40% of the total diet [40]. Taken together, these comparisons indicate that the DON levels detected in Korean commercial pet food are well below all international guideline values.

Furthermore, the DON concentrations observed in this study were generally comparable to those reported in Romania [41], Spain [42], and Italy [43], although some samples in our study showed higher levels. Recently, 34 dry dog food samples acquired from the Romanian market underwent mycotoxin level testing, revealing an average DON value of 429.67 ng g^−1^, with a minimum value of 135.11 ng g^−1^ and a maximum value of 839.21 ng g^−1^ [41]. Macías-Montes et al. [42] monitored dry cat (*n* = 60) and dog (*n* = 62) food from Spain and detected DON in 100% of the samples, with average levels of 78.52 ± 96.51 ng g^−1^ for dog food and 100.92 ± 77.12 ng g^−1^ for cat food. Grandi et al. [43] analyzed 62% of premium cat food and 100% of standard cat food samples collected in Italy in 2015, finding higher contamination levels in the standard category, with levels of 77.7 ± 117 ng g^−1^ in premium cat food and 209 ± 351 ng g^−1^ in standard cat food. Gazzotti et al. [44] analyzed 24 premium and 24 standard dog food samples from Italy, finding DON contamination in all samples, with higher levels in the standard category, 81.3 ± 61.7 ng g^−1^ in premium and 103 ± 75 ng g^−1^ in standard samples.

In our previous monitoring study conducted in the Republic of Korea in 2021, DON was detected in 26.8% of pet food samples (108 out of 403), with a median concentration of 472 ng g^−1^ [45]. In comparison, the contamination frequency observed in the present study was lower.

**Figure 3:**
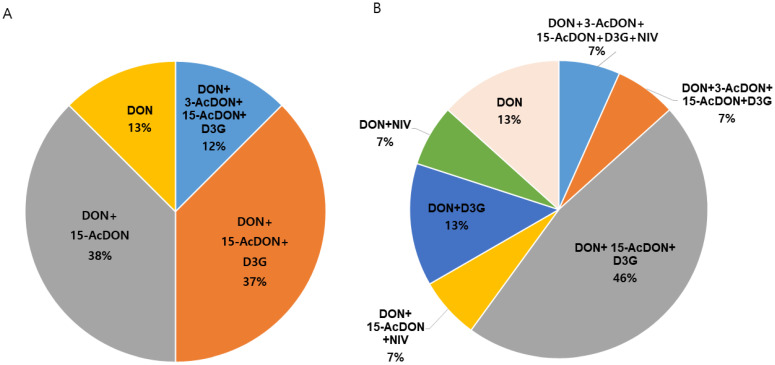
Co-contamination with DON, its derivatives, and NIV was observed in DON-positive cat (**A**) and dog (**B**) food samples. DON, deoxynivalenol; D3G, deoxynivalenol-3-glucoside; ActDON, acylated deoxynivalenol; NIV, nivalenol.

Figure 3 shows the results of co-contamination with DON metabolites (3-AcDON, 15-AcDON, and D3G) and nivalenol (NIV) in dry pet food samples where DON was detected. The most common combinations in cat food were DON+15-AcDON (37.5%) and DON+15-AcDON+D3G (37.5%). The most common combination in dog food was DON+15-AcDON+D3G (46.7%), followed by DON (13.3%) and DON+D3G (13.3%). Co-contamination with DON and its metabolites is more prevalent than DON alone, so continuous monitoring of DON metabolites is required.

EFSA [8] analyzed 10,771 data points reported by 18 European countries between 2007 and 2014. The results for DON and its metabolites in compound feed for livestock and cereal grains, including their derived and subsidiary products, were as follows: Cereal grains and compound feed, primarily barley, maize, oats, and wheat, had average concentrations of 454 ng g^−1^ for DON, 19.0 ng g^−1^ for 3-AcDON, 53.0 ng g^−1^ for 15-AcDON, and 235 ng g^−1^ for D3G. The average concentrations of DON, 3-AcDON, 15-AcDON, and D3G in compound feed were 416, 25.0, 36.0, and 16.6 ng g^−1^, respectively. The CONTAM Panel of EFSA calculated the proportional level ratios of 3-AcDON, 15-AcDON, and D3G concentrations to DON as 10, 15, and 20%, respectively. It highlighted that these findings were consistent with those observed in the feed.

Although the EFSA feed data did not report the quantities of DON and its metabolites in dog and cat food, a comparison with this study showed that, although the detection rates were lower, the detection levels were similar.

Panasiuk et al. [19] reported the incidences of DON, D3G, 3-AcDON, 15-AcDON, and NIV in poultry and pig feed samples (*n* = 99) to be 85, 86, 35, 26, and 23%, respectively. The contamination levels were reported as 10–1709 ng g^−1^ for DON and 1.70–385 ng g^−1^ for D3G. Witaszak et al. [11] investigated pet food samples collected from the Polish market (cat: 12, dog: 26) for the presence of deoxynivalenol (DON) and nivalenol (NIV). The concentrations of DON ranged from 22.2 to 618.4 ng g^−1^ in cat food and 24.5 to 554.3 ng g^−1^ in dog food, while NIV levels ranged from 31.6 to 265.8 ng g^−1^ in cat food and 29.6 to 325.5 ng g^−1^ in dog food.

As shown in Table 5, as the grain content score increased, the DON detection rate in cat food increased from 0 to 6.5 to 25.0%, while that in dog food increased from 2.1 to 7.1 to 14.0 to 25.0%.

The *t*-test results for DON concentrations in the no-grain and grain-containing groups for cat and dog food showed significant differences at the *p* < 0.05 level, with *p* = 0.016 for cat food and *p* = 0.004 for dog food. Various quantities of corn, rice, barley, wheat, sorghum, and grain by-products are commonly used in dry pet food owing to their affordability and relevance as sources of energy, protein, fiber, and other nutrients; however, they are likely to act as mediators of DON contamination. Among the non-grain dog food samples, DON was detected in one occurrence at a 154.3 ng g^−1^ level. Pearson’s correlation analysis was conducted to investigate the relationship between grain content level and the detection rate of deoxynivalenol in cat and dog food. A very strong positive correlation was observed in both species, with r = 0.964 for cat food and r = 0.984 for dog food (*p* < 0.05), indicating that the detection frequency of fusarium-derived mycotoxins increased markedly as the proportion of cereal ingredients increased. These findings suggest that the inclusion of grain-based ingredients in pet food formulations elevates the risk of contamination by Fusarium toxins such as deoxynivalenol.

Witaszak et al. [11] reported DON concentrations of 30.9 ng g^−1^ in grain-free dog food samples, whereas the current study observed concentrations approximately 5-fold higher. The main ingredients of the grain-free dog food in which DON was detected included chicken, chicken meal, sweet potato, tapioca starch, and potato. In grain-free animal feed, cereals are substituted by potatoes and beet pulp [11]. Although DON is primarily found in cereals such as wheat, corn, and oats, contamination has been reported in starchy staple feeds such as potatoes [46,47,48,49]. Therefore, safety assessment and management of pet food should consider cereals and other ingredients.

## 3. Conclusions

This is the first study on the QuEChERS-based LC-MS/MS technique, which was refined to enable the simultaneous quantification of DON, 15-AcDON, 3-AcDON, D3G, and NIV in dry pet food. By applying the sample preparation procedure from the *Animal Feed Standard Analysis Method* of MAFRA in the Republic of Korea, we optimized the LC–MS/MS conditions. The mobile phase was modified to an ammonium acetate system, and the analytical column was replaced with a C18 PFP column, which enhanced ionization efficiency and overall sensitivity. Consequently, the LOQ for DON was reduced from 50 µg/kg to 20 µg/kg, and newly optimized MRM transitions for 3-AcDON, 15-AcDON, D3G, and NIV further demonstrated the technical robustness of the method. Spiking recoveries ranged from 60.1% to 107.2%, with RSDs between 1.7% and 9.3%, satisfying the recovery (60–110%) and precision (RSDr ≤ 20%; RSDR ≤ 40%) criteria specified for DON (>100–≤500 ng g^−1^) in Commission Regulation (EC) No. 401/2006. However, the recoveries of D3G and nivalenol were slightly below the generally recommended threshold of 70%, indicating a remaining limitation of the method.

In our study, strong MEs were observed, indicating the need to use ^13^C-labeled compounds as ISTDs or matrix-matched calibration methods to ensure accurate quantification. The proposed method was used to analyze 246 dry pet food samples commonly consumed in South Korea. The findings indicated that among DON, D3G, 3-AcDON, 15-AcDON, and NIV, DON was detected in 11.8% of cat food and 8.4% of dog food samples, with levels ranging from 122.9 to 799.4 ng g^−1^ in cat food and from 79.7 to 698.0 ng g^−1^ in dog food. These levels are significantly lower than the maximum allowable limit of 5000 ng g^−1^ set by both the EU and MAFRA.

In DON-positive samples, co-contamination of DON and its metabolites was most prevalent in cat food, with DON+15-AcDON and DON+15-AcDON+D3G each accounting for 37.5%, while in dog food, the most common co-contamination was DON+15-AcDON+D3G at 46.7%. A significant difference in the DON contamination levels was observed depending on whether grains were included in dry pet food. As grains are among the primary sources of toxic contaminants in animal feed, continuous monitoring is required. This study can be effectively utilized in quality control laboratories for high-throughput routine analysis of mycotoxins.

## 4. Materials and Methods

### 4.1. Chemicals and Reagents

DON, D3G, 3-AcDON, 15-AcDON, and NIV standards, and ^13^C_15_-DON, ^13^C_17_-3-AcDON, ^13^C_17_-15-AcDON, and ^13^C_21_-D3G isotope-labeled ISTDs were purchased from Cfm Oskar Tropitzsch GmbH (Marktredwitz, Germany). Ultrapure water (18.2 Ω) was obtained using a Milli-Q water purification system (Millipore, MA, USA). Acetonitrile (ACN), methanol (MeOH), and LC-MS-grade water were obtained from Merck (Darmstadt, Germany). Ammonium acetate and acetic acid were purchased from Sigma-Aldrich (St. Louis, MO, USA). Both the pre-weighed QuEChERS salt mixture for extraction, containing 4 g of MgSO_4_ and 1 g of NaCl, and a dispersive-SPE (PSA 25 mg, C18 25 mg) 2 mL tube for purification, containing PSA Bondesil and octadecylsilane (C18), were purchased from BEKOlut GmbH & Co. KG (Hauptstuhl, Germany).

### 4.2. Sample Collection and Preparation

All samples were collected between March and September 2024. This study used extruded dry pet food with a moisture content of <14%. A total of 246 pet food samples (68 cat and 178 dog food samples) of different brands were randomly collected from markets in various locations in South Korea for mycotoxin monitoring. A representative sample (≥500 g) was ground to pass through a standard sieve with a mesh size of 30 mesh (600 μm or smaller). The ground samples that passed through the sieve were mixed to ensure homogeneity. All samples were ground using an SM 300 cutting mill (Retsch, Haan, Germany) to a <4 mm particle size and stored at −20 °C for use.

### 4.3. Sample Extraction and Purification

The homogenized sample (5.0 g) was placed in a 50 mL centrifuge tube. Thereafter, 10 mL of 10% formic acid in water and 10 mL of ACN were added, and the mixture was shaken for 30 min. After shaking, 4 g of anhydrous magnesium sulfate and 1 g of sodium chloride were added to the extract, mixed vigorously for 1 min, and then centrifuged at 4000× *g* for 10 min at 4 °C, after which 1 mL of the supernatant was collected. Subsequently, 1 mL of the supernatant obtained from the extraction was added to a 2 mL centrifuge tube pre-filled with d-SPE (25 mg PSA and 25 mg C18) and mixed thoroughly for 1 min. Next, the mixture was centrifuged at 4000× *g* for 10 min at 4 °C. Next, 400 µL of the purified supernatant was combined with 500 µL deionized water and 100 µL ACN, stirred, and allowed to stand at 4 °C for 30 min. Finally, the solution was filtered through a membrane filter (PTFE, 0.2 μm) to obtain the test solution.

### 4.4. Determination of Mycotoxins Using LC-MS/MS

#### 4.4.1. HPLC Conditions

A Nexera HPLC system (Shimadzu, Kyoto, Japan) and a Triple Quad™ 5500 LC/MS/MS (AB SCIEX, Framingham, MA, USA) were used for mycotoxin analysis. The analytical column was a reverse-phase Mastro2 PFP (150 mm × 2.0 mm, 3.0 μm, Shimadzu, Kyoto, Japan). Water containing 5 mM ammonium acetate and 0.1% acetic acid (A) and methanol/water (95/5:*v*/*v*) with 5 mM ammonium acetate and 0.1% acetic acid (B) were used as the mobile phases. The HPLC separation was performed using a linear gradient program, as summarized in Table 6: 1% B (0–1.5 min), 15% B (1.5–4.0 min), 25% B (4.0–9.0 min), 40% B (9.0–9.1 min), 65% B (9.1–14.0 min), and 1% B (14.1–18.0 min). The total run time was 18.0 min. The flow rate was 0.3 mL min^−1^ at a temperature of 40 °C, and the injection volume was 3 µL.

#### 4.4.2. LC-MS/MS Conditions

A Triple Quad 5500 LC/MS/MS System (AB SCIEX) equipped with electrospray ionization (ESI) was used for mycotoxin detection. MRM was performed in negative and positive modes to identify and quantify mycotoxins. The source and gas parameters were kept constant throughout the acquisition: source temperature 400 °C, curtain gas 45 psi, CAD gas 12, ion source gas 1:50 psi, and ion source gas 2:50 psi. The positive- and negative-polarity spray voltages were set to 5000 and 4500 V, respectively.

### 4.5. Evaluation of MEs

The ME caused by the sample was evaluated using ISTD and matrix-matched quantification methods. The solvent calibration curve was evaluated by preparing five standard solutions in the 10–500 ng g^−1^ range in ACN/water (1:1, *v*/*v*). The ISTD calibration curve was constructed based on the solvent calibration curve by adding 2 μL of ISTD in the same concentration range. The slopes of both sets of calibration curves were determined by plotting the concentration against the peak area using linear regression analysis and then comparing them. The percentage ME was calculated using the following two equations [33]:

% ME = 100 − (100 × slope of spiked matrix extract/slope of solvent standard),(1)

% ME = 100 − (100 × slope of ISTD calibration curve/slope of solvent standard),(2)

Positive matrix effect values indicate ion suppression, while negative values indicate ion enhancement; according to SANTE/12089/2016, matrix effects within ±20% are considered negligible [35].

### 4.6. Qualitative and Quantitative Assessment of Mycotoxins

A qualitative analysis was performed by comparing the retention times of the peaks in the chromatogram with those of the standard solution. Additionally, the precursor and product ions of both the standard and test solutions were matched, and the response ratio between the product ions of the standard and test solutions was compared to ensure that the ratio was within ±30%.

For quantitative analysis using ISTDs, calibration curves for each standard were constructed from the peak area ratios of the standard and ISTD. Quantification was performed based on the peak area ratios of the quantitative ions from the chromatogram of the test solution and the quantitative ions of the ISTD. An ISTD was added by mixing it with the solution before injection using an autosampler program. The addition levels of the isotope-labeled ISTDs (^13^C_15_-DON, ^13^C_17_-3-AcDON, ^13^C_17_-15-AcDON, and ^13^C_21_-D3G) were all 100 μg L^−1^, and the injection volume was 2 μL.

For the matrix-matched calibration with quantitative analysis, a calibration curve was prepared under the same conditions as the sample pretreatment by mixing 0.4 mL of the purified supernatant from blank pet food samples, 0.5 mL of distilled water, and 0.1 mL of standard solutions (100, 500, 1000, 2500, and 5000 ng g^−1^) dissolved in acetonitrile. Quantification was performed based on the peak area obtained under the same conditions as those used for the qualitative analysis. The blank pet food sample extract was prepared by extracting and purifying a sample of the same type as the test sample. Still, it did not contain detectable mycotoxin components, adhering to the steps outlined in Section 4.2 and Section 4.3.

### 4.7. Method Validation

The validation parameters of the established method, including linearity, LOD, LOQ, accuracy, intermediate precision (reproducibility), and repeatability, were evaluated according to the guidelines of MAFRA [37] and SANTE/11813/2017 [35]. Linearity was determined by measuring five concentrations in the dog and cat food samples, including the LOQ and expected concentrations. Linearity was applied using the correlation coefficient (*R*^2^), *y*-intercept, slope of the regression line, and sum of the squared residuals. The LOD was determined by adding the lowest validated concentration that met the recovery criteria according to SANTE/11813/2017 to dog and cat food samples in which mycotoxins were not detected, followed by seven repetitions of the measurements.

A calibration curve was constructed by measuring the mycotoxins across five concentrations, starting from the lowest concentration. The calibration curve was expressed as a linear equation, *y* = *a* + *bx* (where *a* is the *y*-intercept, *b* is the slope, and *x* is the concentration). The slope (S) represents the slope of the linear equation. The standard deviation (σ) is determined from the area of measurements taken at approximately twice the LOQ concentration, repeated seven times.

To evaluate accuracy, a mixed standard solution was added to the blank pet food sample at three concentrations (LOQ, LOQ × 2, and LOQ × 5). The experiment was repeated thrice for each concentration, and the recovery rate and RSD were calculated. The method’s precision was assessed in two steps: repeatability was evaluated with at least six replicate measurements of the entire procedure using a blank extract spiked at a concentration corresponding to 100% of the test concentration. Second, we evaluated reproducibility using the same concentration over three separate days, with measurements taken thrice daily, and then the RSDs were calculated.

### 4.8. Feed Grouping Based on Grain Content

According to the FDA’s FY21 Aflatoxin Surveillance Report, a trend was observed in which the likelihood of aflatoxin positivity increased when grains were listed higher in the pet foods’ ingredient list; this suggests that the order in which grains appear in the ingredient list may serve as a meaningful indicator of the risk of mycotoxin contamination [50]. Based on this evidence, the present study estimated the relative grain content by assigning scores to the position of grains in the pet foods’ ingredient list. Pet foods were then grouped to analyze the correlation between mycotoxin contamination and grain content. The grouping by grain content was done by scoring the relative grain amount based on the number and order of grain ingredients listed. Pet foods with no grain ingredients (score of 0) were classified into the ‘No grain’ group. Scores ranging from 1 to 15 were assigned depending on the number and position of grain components. Based on these scores, pet foods were categorized into four groups to reflect the degree of grain inclusion: 0 points (No grain), 1–5 points (Low grain), 6–10 points (Medium grain), and 11–15 points (High grain).

### 4.9. Data Analysis

All analytical data acquisition and processing were performed using SCIEX OS (version 3.3.1.43; AB SCIEX). Microsoft Excel 2013 (Microsoft Co., Redmond, WA, USA) was used to calculate the median values of the experimental data and conduct *t*-tests.

## Figures and Tables

**Table 1:** Analytical conditions for quantifying deoxynivalenol, its derivatives, and nivalenol and related isotope-labeled compounds in ESI(−) and ESI(+) using the UPLC-MS/MS method.

Analytes	Adduct	Retention Time (min)	Precursor Ion (*m*/*z*)	Product Ion (*m*/*z*)	DP (V)	EP (V)	CE (eV)	CXP (V)
DON	[M + CH_3_COO]^−^	8.99	355	295.1	−50	−10	−14	−10
				59	−50	−10	−50	−10
D3G	[M + CH_3_COO]^−^	9.40	517.1	427.1	−30	−10	−30	−10
				457	50	10	60	10
NIV	[M + CH_3_COO]^−^	6.58	371.1	281.1	−50	−10	−22	−13
				59	−50	−10	−50	−11
3-AcDON	[M + CH_3_COO]^−^	13.30	397.3	337.1	−50	−10	−13	−10
				307.2	−50	−10	−40	−10
15-AcDON	[M + NH4]^+^	13.11	356	321.1	25	10	20	10
				137	25	10	21	10
^13^C_15_-DON	[M + CH_3_COO]^−^	8.99	370	279	−50	−10	−14	−10
^13^C_21_-D3G	[M + CH_3_COO]^−^	9.40	538	478	−70	−10	−20	−10
^13^C_17_-3-AcDON	[M + CH_3_COO]^−^	13.30	414	354	−70	−10	−15	−10
^13^C_17_-15-AcDON	[M + NH4]^+^	13.11	373	338	30	10	20	10

ESI, electrospray ionization; UPLC-MS/MS, DON, deoxynivalenol; D3G, deoxynivalenol-3-glucoside; ActDON; acylated deoxynivalenol NIV; nivalenol; DP, declustering potential; EP, entrance potential; CE, collision energy; CXP, collision cell exit potential.

**Table 2:** Limit of detection, limit of quantification, linear range, and linearity for five mycotoxins (DON, D3G, 3-AcDON, 15-AcDON, and NIV) in dog and cat food sample, analyzed by LC-MS/MS using a pentafluorophenyl (PFP) column and a mobile phase consisting of water (A) and methanol/water(95/5:*v*/*v*) (B), both containing 5 mM ammonium acetate and 0.1% acetic acid.

B-Type C-TCNs	Matrix	r^2^	LOD (ng/g)	LOQ (ng/g)	Precision (% RSD)	
					Intra-Day (n = 6)	Interday (n = 9)
DON	Dog	0.99816	8.3	24.8	3.2	5.9
	Cat	0.99848	9.4	28.1	2.7	4.5
D3G	Dog	0.99543	8.5	25.5	8.4	8.9
	Cat	0.99590	7.7	23.1	7.4	8.3
3-AcDON	Dog	0.99991	8.5	25.6	2.9	5.8
	Cat	0.99509	7.9	23.8	5.1	9.6
15-AcDON	Dog	0.99941	6.9	20.6	3.7	9.3
	Cat	0.99648	6.7	20.1	2.9	9.4
NIV	Dog	0.99971	8.6	25.9	5.6	6.3
	Cat	0.99888	7.4	22.0	3.2	5.9

LC-MS/MS, liquid chromatography quadrupole mass spectrometry; DON, deoxynivalenol; D3G, deoxynivalenol-3-glucoside; ActDON, acylated deoxynivalenol; NIV, nivalenol; LOD, limit of detection; LOQ, limit of quantification; RSD, relative standard deviation.

**Table 3:** Results of the Association of American Feed Control Officials (AAFCO) proficiency test for dry cat food matrix, analyzed by LC-MS/MS. Analytical conditions are described in Section 4.4.

Analyte	DON ISTD (ng g^−1^)			DON Matrix-Matched (ng g^−1^)		
AAFCO (2023-62 dry cat feed)	Samples	Average	*Z*-Score	Samples	Average	*Z*-Score
	2013.9	2017.2 *	0.78–0.79	2072.5	2033.1 *	0.63–0.78
	2020.2			1938.4		
	2017.6			2088.4		

* According to the *t*-test results *(p > 0.05).* DON, deoxynivalenol; ISTD, internal standard.

**Table 4:** Monitoring DON, its modified forms, and NIV in dog and cat food.

Matrix	Total No.	Mycotoxins	No. of Positive Samples (%)	Concentration (ng g^−1^)		
				Minimum	Maximum	Median
Cat	68	DON	8(11.8%)	122.9	799.4	268.9
		D3G	5(7.4%)	45.3	141.9	65.7
		3-AcDON	2(2.9)	29.2	33.8	31.5
		15-AcDON	6(8.8%)	33.8	101.6	58.2
		NIV	N.D.	<LOQ	<LOQ	<LOQ
Dog	178	DON	15(8.4%)	79.7	698.0	317.5
		D3G	11(6.2%)	27.2	97.5	77.0
		3-Act DON	2(1.1%)	23.8	24.6	24.2
		15-AcDON	10(5.6%)	22.5	207.9	65.0
		NIV	3(1.7%)	23.4	32.0	29.8

No., number of total tested samples; N.D., not detected; LOQ, limit of quantification; DON, deoxynivalenol; D3G, deoxynivalenol-3-glucoside; ActDON, acylated deoxynivalenol; NIV, nivalenol.

**Table 5:** Detection rate and concentrations of DON according to grain content.

**Cat**						
	No grain	Low grain	Medium grain	High grain	Pearson’s correlation coefficient (r)	Total number of samples
Score	0	1–5	6–10	11–14	0.964	68
Detection rate	0/12(0%)	2/31(6.5%)	6/25(24.0%)	-		
Concentration range(ng g^−1^)	<LOQ *	122.9–162	178.5–799.4	-		
**Dog**						
	No grain	Grain			Pearson’s correlation coefficient (r)	Total number of samples
Score	0	1–5	6–10	11–14	0.984	178
Detection rate	1/47(2.1%)	5/70(7.1%)	8/57(14.0%)	1/4(25.0%)		
Concentration range(ng g^−1^)	154.3 *	79.7–545.1	106.2–698	121		

* *p* value < 0.05. LOQ, limit of quantification.

**Table 6:** The mobile phase gradient for separation of mycotoxins.

Time (min)	Flow (mL/min)	A%	B%
1.5	0.3	99.0	1.0
4.0	0.3	85.0	15.0
9.0	0.3	75.0	25.0
9.1	0.3	60.0	40.0
14.0	0.3	35.0	65.0
14.1	0.3	99.0	1.0
18.0	0.3	99.0	1.0

## Data Availability

The original contributions presented in this study are included in the article/Supplementary Materials. Further inquiries can be directed to the corresponding authors.

## Supplementary Materials

The following supporting information can be downloaded at: https://www.mdpi.com/article/10.3390/toxins17120590/s1, Figure S1: Comparison of matrix effects on mycotoxin quantification using a solvent-based standard curve versus an internal standard (ISTD)-based calibration in cat food. The blue line represents the ISTD-based standard curve, and the orange line represents the solvent-based standard curve; Figure S2: Comparison of matrix effects on mycotoxin quantification using a solvent-based standard curve versus an internal standard (ISTD)-based calibration in dog food. The blue line represents the ISTD-based standard curve, and the orange line represents the solvent-based standard curve; Figure S3: Comparison of matrix effects on mycotoxin quantification using a solvent-based standard curve versus a cat food matrix-matched calibration. The blue line represents the matrix-matched standard curve, while the orange line represents the solvent-based standard curve; Figure S4: Comparison of matrix effects on mycotoxin quantification using a solvent-based standard curve versus a dog food matrix-matched calibration. The blue line represents the matrix-matched standard curve, while the orange line represents the solvent-based standard curve. Table S1. Matrix effects (%) of five mycotoxins in dog and cat food matrices using matrix-matched calibration and ISTD-based quantification under ESI(−) and ESI(+) modes. Table S2. Signal enhancement factors when neither matrix-matched nor ISTD calibration is applied. Table S3. International guideline or regulatory levels for deoxynivalenol in dog and cat food.

## Abbreviations

The following abbreviations are used in this manuscript:

EC	European Commission
EFSA	European Food Safety Authority
ESI	Electrospray Ionization
MAFRA	Ministry of Agriculture, Food and Rural Affairs
DON	Deoxynivalenol
D3G	Deoxynivalenol-3-glucoside
3-AcDON	3-Acetyl-Deoxynivalenol
15-AcDON	15-Acetyl-deoxynivalenol
NIV	Nivalenol
RSD	Relative Standard Deviations
MEs	Matrix Effects
MRM	Multiple Reaction Monitoring
PSA	Primary Secondary Amine
