# Supplementary material for: Concurrent Quantification of Deoxynivalenol, Its Derivatives, and Nivalenol in Pet Food Using QuEChERS Combined with LC-MS/MS
Source: Toxins (Basel). 2025 Dec 10;17(12):590. doi: 10.3390/toxins17120590 (PMC12737684; doi:10.3390/toxins17120590)
Supplement: Supplementary file 1 [file toxins-17-00590-s001.zip › toxins-3864305-supplementary.pdf]

toxins

MDPI

Supplementary Materials: Concurrent Quantification of Deoxynivalenol, Its Derivatives, and Nivalenol in Pet Food Using QuEChERS Combined with LC-MS/MS

Chae-Eun Yeo, Subin Gwon, Eun Hee Chang, Hyo Young Kim, Sung-Youn Kim, Kangmin Seo, Ji Hye Lee and Hyunjeong Cho

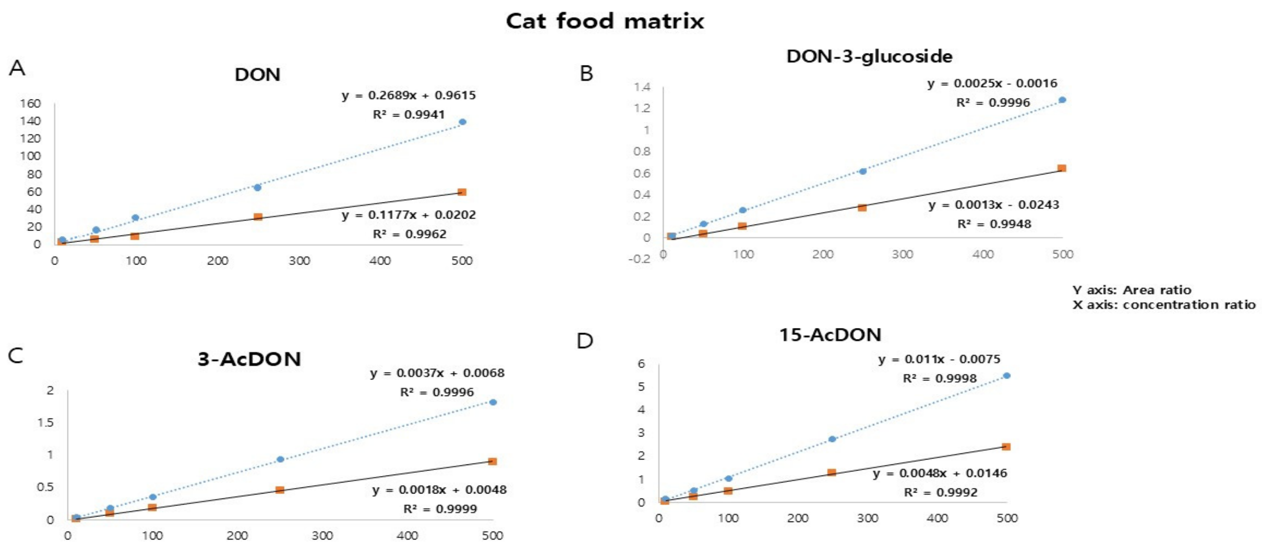

Figure S1. Comparison of matrix effects on mycotoxin quantification using a solvent-based standard curve versus an internal standard (ISTD)-based calibration in cat food. The blue line represents the ISTD-based standard curve, and the orange line represents the solvent-based standard curve.

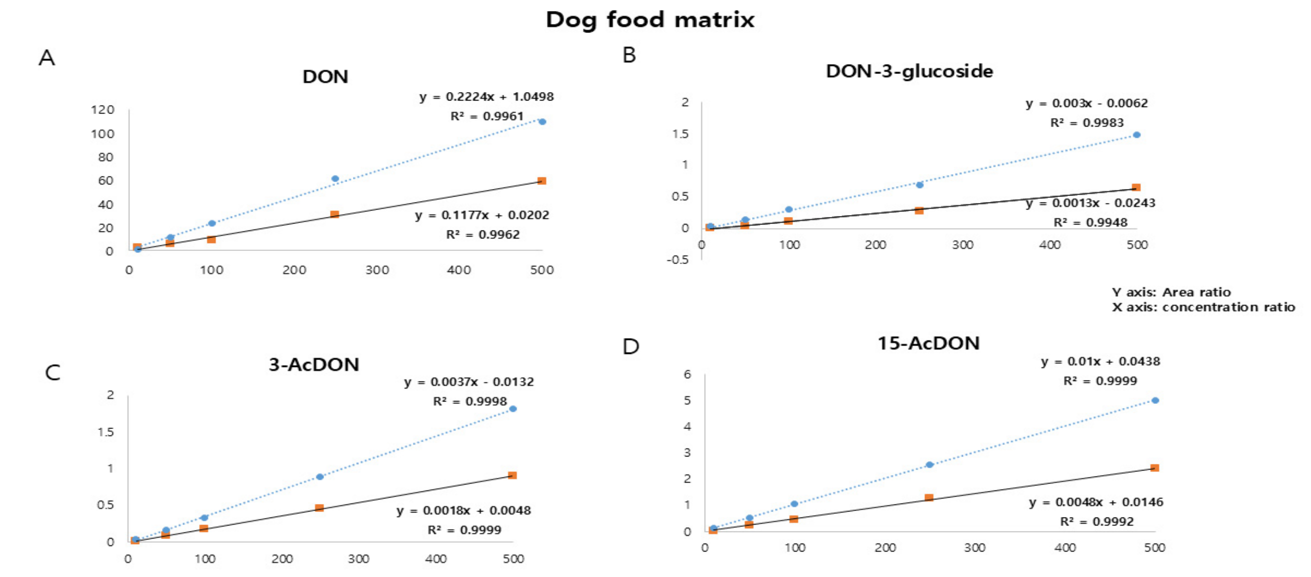

Figure S2. Comparison of matrix effects on mycotoxin quantification using a solvent-based standard curve versus an internal standard (ISTD)-based calibration in dog food. The blue line represents the ISTD-based standard curve, and the orange line represents the solvent-based standard curve.

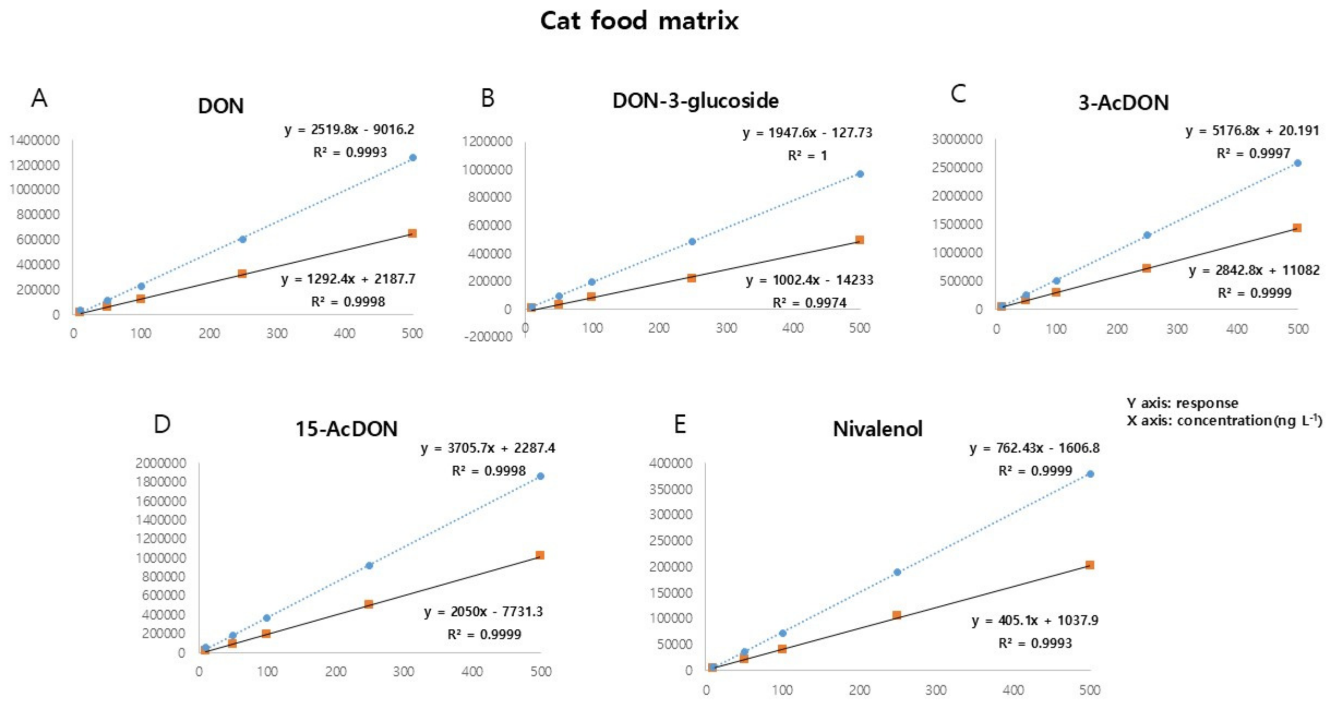

Figure S3. Comparison of matrix effects on mycotoxin quantification using a solvent-based standard curve versus a cat food matrix-matched calibration. The blue line represents the matrix-matched standard curve, while the orange line represents the solvent-based standard curve.

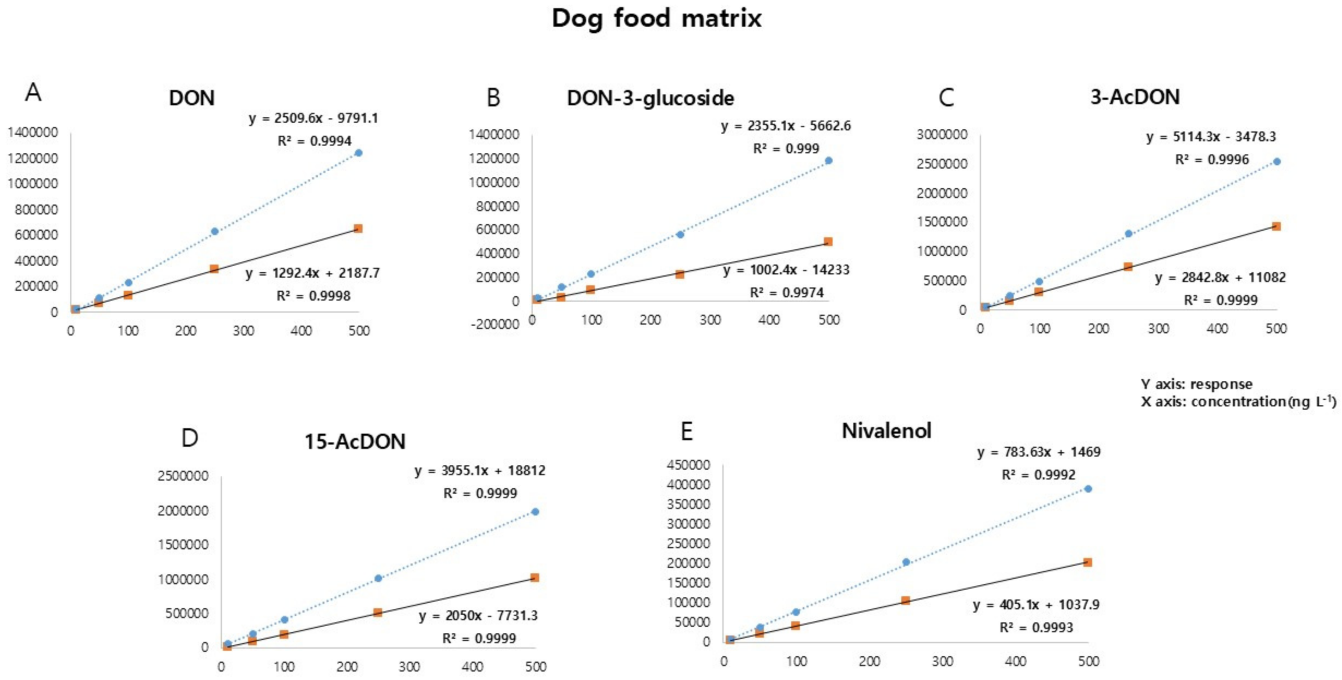

Figure S4. Comparison of matrix effects on mycotoxin quantification using a solvent-based standard curve versus a dog food matrix-matched calibration. The blue line represents the matrix-matched standard curve, while the orange line represents the solvent-based standard curve.

Table S1. Matrix effects (%) of five mycotoxins in dog and cat food matrices using matrix-matched calibration and ISTD-based quantification under ESI(−) and ESI(+) modes.

Mycotoxins	Ionization mode	Matrix matched calibration curve		ISTD curve	
		Dog	Cat	Dog	Cat
DON	ESI-	-94.2%	-95.0%	-88.9%	-128.5%
D3G	ESI-	-134.9%	-94.3%	-130.8%	-92.3%
3-AcDON(-)	ESI-	-79.9%	-82.1%	-105.6%	-105.6%
NIV	ESI-	-93.4%	-88.2%	-	-
15-AcDON(+)	ESI+	-96.2%	-108.8%	-108.3%	-129.2%

Table S2. Signal enhancement factors when neither matrix-matched nor ISTD calibration is applied.

Toxins	Ionization mode	Matrix matched calibration curve		ISTD cuvrve	
		Dog	Cat	Dog	Cat
DON	ESI-	1.9	2.0	1.9	2.3
D3G	ESI-	2.4	1.9	2.3	1.9
3-AcDON	ESI-	1.8	1.8	2.1	2.1
NIV	ESI-	1.9	1.9	-	-
15-AcDON	ESI+	**2.0**	**2.1**	2.1	2.3

Table S3. International guideline or regulatory levels for deoxynivalenol in dog and cat food.

Nation	Commodity	Limit (ng g^{-1})	Type of standard
EU	compound feed for calves (< 4 months), lambs, kids and dogs	2,000	guideline level
Japan	Dog feed	2,000	legislated compositional standard
	Cat feed	1,000	
Korea	Compound feed (excluding premix feed)	5,000	guideline level
US	grain and grain byproducts(not to exceed 40% of the diet) for all other animals	5,000	advisory level
